# Digestion-resistant maltodextrin effects on colonic transit time and stool weight: a randomized controlled clinical study

**DOI:** 10.1007/s00394-015-1045-4

**Published:** 2015-10-06

**Authors:** María Salud Abellán Ruiz, María Dolores Barnuevo Espinosa, Carlos J. Contreras Fernández, Antonio J. Luque Rubia, Francisca Sánchez Ayllón, Miriam Aldeguer García, Carlos García Santamaría, Francisco Javier López Román

**Affiliations:** 1 Cátedra de Fisiología del Ejercicio. Facultad de Ciencias de la Salud, UCAM -Universidad Católica de Murcia, Campus de los Jerónimos, n° 135 Guadalupe, 30107 Murcia, Spain; 2 Facultad de Enfermería, Universidad Católica San Antonio, Murcia, Spain

**Keywords:** Colonic transit time, Resistant maltodextrin, Soluble dietary fibre, Stool volume, Intestinal function

## Abstract

**Purpose:**

Increased awareness of the importance of dietary fibre has led to increased interest in “functional” fibre components like digestion-resistant maltodextrin (RMD). This randomized, placebo-controlled, double-blind study assessed the effects of RMD in the colonic transit time (CTT) and defecation characteristics (frequency, stool volume and consistency).

**Methods:**

Sixty-six healthy adult volunteers (32 men) who did not have a daily defecation habit had a 7-day run-in period before the 21-day intervention period with RMD or placebo. CTT and segmental CTT (SCTT) were assessed by a single abdominal X-ray film taken at the end of both periods after radiopaque marker ingestion. Defecation characteristics and intestinal functions were also assessed, which were self-reported by patients. Intragroup comparisons were evaluated by Student’s paired *t* test, Bonferroni test and Chi-square test, while time comparisons by analysis of variance (ANOVA) and time-by-treatment interaction by repeated-measures ANOVA.

**Results:**

Fifty-seven subjects were assessed for CTT (placebo, *n* = 28; RMD, *n* = 29). In the RMD group, the total CTT, left SCTT and rectosigmoidal SCTT decreased significantly compared to baseline (*p* < 0.01 each; −13.3, −4.7, −8.7 h, respectively). Significant differences between groups were observed in total CTT and left SCTT. Significant time-by-treatment interaction was observed in the RMD group for stool volume (*p* = 0.014), increasing 56 % compared to baseline (*p* < 0.01), while remained unchanged in the placebo group. Stool consistency was improved only in the RMD group (*p* < 0.01). No adverse effects related to study products were observed.

**Conclusions:**

The results show that RMD improved CTT, stool volume, stool consistency and some intestinal functions in a healthy population.

## Introduction

Food processing has undoubtedly had a permanent effect on Western eating habits and led to an unparalleled reduction in dietary fibre (DF) consumption [1, 2]. The FAO and WHO recommend an adequate intake of total fibre of 38 g for men and 25 g for women [3, 4]. According to the European Food Safety Authorization (EFSA), a daily intake of 25 g of fibre per day is adequate for normal laxation in adults [5]. However, and following FAO/WHO recommendations [3], in the USA, a balanced diet is recommended to contain between 25 and 38 g of DF [6], but in reality this figure is rarely met as DF consumption is on average only 40 % of the recommended dietary allowance [7]. The DF can be defined from multiple points of view, as reviewed by Fuentes-Zaragoza et al. [4]. The Codex Alimentarius Commission’s Committee on Nutrition and Foods for Special Dietary Uses defined DF as “carbohydrate polymers with 10 or more monomeric units, which are not hydrolysed by the endogenous enzymes in the small intestine of humans” [8], definition that included resistant starch, oligosaccharides and other non-digestible carbohydrates [4]. The importance of increasing DF consumption in the general population has both health and economic benefits as it has been shown to be inversely associated with many chronic diseases such as coronary heart disease [9], certain cancers [10], diabetes [11] and obesity [12]. Furthermore, a fibre deficiency can result in a number of associated digestive and metabolic disorders such as inflammatory bowel disease, metabolic syndrome and impeded faecal transit time [6, 13, 14]. In order to combat the increasing prevalence of these disorders, “functional” fibre components, namely non-digestible carbohydrates that have beneficial physiological effects in humans, have been added to a wide variety of foods. For instance, the intake of a dairy preparation with a fibre supplement containing 20 g of soluble fibre improves chronic constipation [15].

In recent years, an increased awareness of the importance of DF has led to increased interest in functional DFs that are neither hydrolysed nor absorbed in the small intestine and pass into the large intestine [16]. To obtain beneficial health benefits is recommended an intake of 20 g/day of resistant starch (RS), a source of dietary fibre. The principal sources of RS are: whole/partly milled grains, seeds, legumes, potatoes, green bananas, high-amylose corn, bread and processed foods in which modified starches been used. However, its dietary intake can vary considerably between countries. For instance, intakes in the EU range from 3 to 6 g/day, in the UK resistant starch intakes are estimated to be 2.76 g/day, and in Sweden is estimated to be 3.2 g/day (reviewed in [17]). The RS food source varies depending on the RS type (reviewed in [17]). Resistant maltodextrin is obtained from corn starch through heat and enzymatic treatment. Thus, this process is comprised of a hydrolysis reaction by heat and hydrochloric acid in low humidity conditions, hydrolysis reaction with amylase, refinement and spray-dried [18, 19]. Digestion-resistant maltodextrin (RMD [18, 20, 21]) is a non-viscous soluble DF, non-digestible carbohydrate and has been reported to have various physiological functions in humans. Intestinal regularity is typically influenced by DF, and studies have shown that RMD increases stool frequency and volume in humans [22]. Furthermore, it has been reported that RMD is fermented by the intestinal bacterial flora, including bifidobacteria, resulting in an increase in the types and number of bacteria in the intestinal flora [20, 23–27]. Fermentation by gut bacteria leads to short-chain fatty acid (SCFA) production, mainly: acetate, propionate and butyrate. For instance, butyrate is one of the SCFA for colonic health due to its effects on promoting normal development of colonocytes. Moreover, the production of SCFA can also lower the luminal pH, which can cause the inhibition of potentially pathogenic bacteria growth [28, 29]. In clinical studies, RMD supplementation has been shown to be highly tolerated. Moreover, RMD has been shown to improve metabolic syndrome by reducing visceral fat and improving glucose and lipid metabolism in humans [14]. Although the effect of RMD to reduce the colonic transit time (CTT) has been confirmed in animal model studies, no human study has been reported [30].

In this context, the main aim of this study was to assess the efficacy of RMD supplementation in reducing the CTT in healthy subjects with Western diet, compared to non-digestion-resistant maltodextrin as placebo. We also evaluated the efficacy in reducing the segmental CTT (SCTT), improving defecation characteristics (frequency, stool volume and consistency) and intestinal functions.

## Methods

### Participants

Healthy participants aged between 18 and 30 years were recruited by advertisements at the Universidad Católica San Antonio de Murcia. Participants had a body mass index (BMI) of less than 30 kg/m^2^ and a physical activity of less than two times per week. Likewise, they did not have a daily defecation habit, history of any digestive disease nor gastrointestinal/abdominal surgery. All participants provided informed consent and understood and fulfilled all the procedures and requirements of the study. Subjects with diabetes, pregnancy and any other conditions that the investigator regarded as unfit for the study were excluded.

### Design

A randomized, placebo-controlled, double-blind, single-centre clinical study was performed with two parallel groups: placebo (non-digestion-resistant maltodextrin) or RMD (Fibersol-2, digestion-resistant maltodextrin, Matsutani Chemical Industry Co., Ltd; [18, 20, 21]) at the Universidad Católica San Antonio de Murcia, Spain. The study protocol and the informed consent form were approved by the Independent Ethics Committee of the Universidad Católica San Antonio de Murcia. The total study duration was 28 days, including a 7-day run-in period before intervention to collect baseline data of participants. This was followed by a 21-day intervention period from day 8 to 28 where the participants consumed one of the allocated study products daily. Medications or other treatments that would result in a change in colonic transit were not permitted, as well as medication or nutritional supplement that interfered with the formulation.

Before and after the intervention period, physical examination, blood sample collection and a questionnaire survey of intestinal functions were conducted. Daily defecation habits, defecation frequency, stool volume and consistency, and 5-day dietary survey in each period were recorded by each subject. Medical history and adverse events (AEs) were checked at the end of each period. CTT and SCTT were assessed by radiography on days 7 and 28.

Subject randomization was performed using a random number generator (www.random.org) that allocated the subjects in each group.

### Interventions

RMD and placebo had the same appearance (in colour and size) and flavour and were provided in a 15-g sachet in powder form to be dissolved in water. Subjects were instructed to consume one sachet daily with breakfast and to maintain their normal diet throughout the study.

### Measurements

#### Colonic transit time

To determine CTT, subjects ingested a capsule containing 24 radiopaque makers (Colognost^®^, Iberoinversa Pharma) daily for five consecutive days at 24-h intervals. Twenty-four hours after the final marker ingestion an abdominal X-ray was performed. To calculate CTT, the time between each marker ingestion (Δ*t*), the number of markers observed on the X-ray (*n*) and the number of markers ingested (*N*) were used in the following formula [31]: CTT (h) = (Δ*t* × *n*)/*N*.

X-rays were performed using a high kilovoltage and low exposure time technique to ensure that marginalized areas of the colon were included in the X-ray field. Two physicians counted the markers independently to minimize interobserver variability. Subsequently, total CTT and SCTT were calculated for each individual using the average from both counts. To segment the colon, the fifth lumbar vertebra (L5) served as central point to draw three imaginary lines: a central line from the third lumbar vertebra (L3) to L5, a right line from the L5 to the right femoral head (RFH) and a left line from the L5 to the left ilium (LI). The area above the right line (L3L5–RFH) was assigned to the right colon, the area above the left line (L3L5–LI) was assigned to the left colon, and the area below and between right and left line (IL–RFH) was assigned to the rectosigmoidal colon.

#### Intestinal function endpoints

Defecation frequency was self-reported. Stool consistency was determined by each subject using the Bristol Stool Chart, which has a 7-point scale from Type 1 for the hardest lumpy stools representing constipation to Type 7 for the watery stools representing diarrhoea. Ideal stool consistency covers scores from 3 to 4. Stool volume was recorded by subjective visual estimation according to predefined comparative object. Subjects were trained to compare and to assess their stool volume converting it into a ping pong ball. Later, they reported to the UCAM roughly the number of ping pong balls they converted. Clinical variables of intestinal function were assessed according to the Rome III Criteria: straining, lumpy or hard stools, sensation of incomplete evacuation, sensation of anorectal obstruction and/or blockage, and manual maneuvers to facilitate defecation during at least 25 % of defecations, as well as to have fewer than three defecations per week. A higher Rome III score correlates with higher functional constipation.

#### Assessment dietary fibre intake

Dietary fibre intake was assessed using a dietary survey. A five-day food record (qualitative and quantitative total intake) that was maintained by the subjects was processed using Diet source^®^ 3.0 that analysed nutritional variables including fibre consumption, energy consumption, macronutrient intake as well as any liquid ingested to ensure that there were no changes in any study phase in both groups. Subjects were advised that they should not vary their dietary habits during the study. Moreover, during marker ingestion the subjects visually recorded each meal. It was checked and verified that female subjects had the same estrogenic conditions and in no period of menstruation in the days of ingestion of radiopaque markers and measurement of colonic transit time by abdominal radiography.

#### Safety

The safety profile of the study products was assessed through the record of adverse events (AEs) and assessment of biochemical parameters.

Blood samples were taken before and after the intervention to asses liver function (ALT, AST) and renal function (urea and creatinine). Subjects were instructed to report any of suspicious reactions, which were evaluated by the medical staff of the Hospital Virgen de la Vega de Murcia as possible AEs.

### Statistical analysis

The sample size was calculated in order to achieve the primary objective, with a confidence level of 95 %, beta error of 80 %, and estimated difference and standard deviation (SD) for the CTT of 5 h and 8.3, respectively [32]. The targeting number required for enrolment was 33 in each group.

Results were expressed as mean ± SD. The colonic transit time and the colonic segmental transit time were compared using ANCOVA for repeated measurements with one intrasubject factor (trial time), one intersubject factor [digestion-resistant maltodextrin (RMD) consumption, with or without consumption] and one covariable (fibre consumption). Faecal frequency, stool consistency and haematological and biochemical variables were compared using ANOVA for repeated measurements with one intrasubject factors (trial time) and one intersubject factor [digestion-resistant maltodextrin (RMD) consumption, with or without consumption]. For the comparison within a group, Dunnett’ multiple comparison test was used. Clinical variables of intestinal function (Roma III criterion) were compared using the Chi-square test. All statistical analysis was performed using SPSS (version 21.0), and a *p* value <0.05 was considered statistically significant.

## Results

### Baseline data

**Fig. 1:**
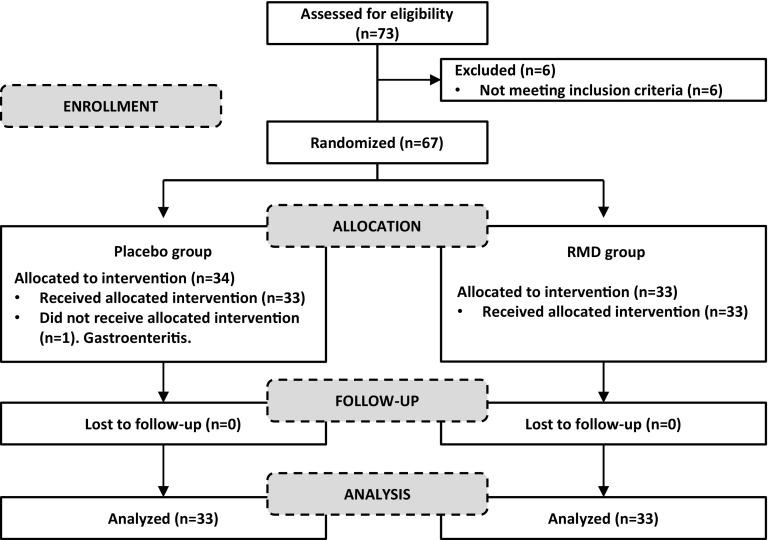
Disposition of subjects for placebo (maltodextrin) and digestion-resistant maltodextrin (RMD) groups

A total of 73 candidates were assessed for eligibility, and 67 subjects met the criteria and were randomly allocated into two groups. One subject was withdrawn during the run-in period before receiving intervention (Fig. 1). Thus, a total of 66 subjects were analysed, 33 in each study group. The demographic and other baseline characteristics of subjects in each group are outlined in Table 1. Briefly, the population was 51.5 % female with the mean age of 21.3 years and the mean BMI 23.0 kg/m^2^. The two study groups were well balanced with respect to demographics and baseline characteristics, and no significant difference was found in any of the variables between groups. The mean daily fibre intake was 10.0 ± 4.6 g in the RMD group and 11.5 ± 4.6 g in the placebo group. Overall, no significant differences in the diet were observed between groups.

**Table 1:** Demographics and baseline characteristics of subjects

	Placebo (*n* = 33)	RMD (*n* = 33)	Total (*n* = 66)
Female, *n* (%)	17 (51.5)	17 (51.5)	34 (51.5)
Age (years)^a^	21.5 ± 3.2	21.1 ± 2.4	21.3 ± 2.8
Height (m)^a^	1.73 ± 0.1	1.72 ± 0.1	1.7 ± 0.1
Weight (kg)^a^	70.3 ± 14.0	67.5 ± 12.3	69.0 ± 13.1
BMI (kg/m^2^)^a^	23.3 ± 3.0	22.7 ± 3.0	23.0 ± 3.0
Smoker, *n* (%)	10 (30.3)	8 (24.2)	18 (27.3)

*RMD* digestion-resistant maltodextrin, *m* metres, *kg* kilograms, *BMI* body mass index, *n* number of subjects

^a^Values expressed as mean ± SD (standard deviation)

### Colonic transit time

Although 66 subjects completed the study, 9 subjects (5 in placebo and 4 in RMD groups) failed to complete CTT determination due to non-compliance. Consequently, data from 57 subjects were used to measure CTT and SCTT.

The total CTT, left SCTT and rectosigmoidal SCTT were significantly decreased in the RMD group after intervention compared to baseline (*p* < 0.004, *p* < 0.008 and *p* < 0.006, respectively), while no decrease was found in the placebo group. There are significant differences observed between groups regarding the total CTT and left SCTT (*p* < 0.028 and *p* < 0.001, respectively; Table 2). Placebo intervention did not yield any decrease for the total CTT and SCTT. Additionally, stratified analysis by gender did not show differences between placebo and RMD groups for CTT and SCTT (data not shown).

**Table 2:** Change in total, right, left and rectosigmoidal colonic transit time for each group after 3-week intervention

Colonic transit time (h)	Treatment	Baseline	Week 3	*Δ*
Total colon	RMD	53.0 ± 23.9	39.7 ± 22.3**	−13.3 ± 21.6^#^
	Placebo	48.4 ± 24.1	48.0 ± 25.0	−0.4 ± 21.4
Right colon	RMD	12.3 ± 8.0	12.3 ± 8.7	0.1 ± 9.6
	Placebo	12.6 ± 12.8	12.4 ± 9.4	−0.2 ± 10.7
Left colon	RMD	14.9 ± 9.9	10.2 ± 8.6**	−4.7 ± 8.8^##^
	Placebo	9.4 ± 9.1	12.1 ± 9.5*	2.7 ± 6.5
Rectosigmoidal colon	RMD	25.9 ± 18.1	17.2 ± 14.6**	−8.7 ± 15.4
	Placebo	26.5 ± 13.1	23.6 ± 16.0	−2.9 ± 18.7

Values expressed as mean ± SD (standard deviation)

*Δ*: change from baseline to week 3

* *p* < 0.05; ** *p* < 0.01, intragroup significant difference (Student’s paired *t* test)

^#^ *p* < 0.05; ^##^ *p* < 0.01, intergroup significant differences (ANOVA)

### Intestinal function endpoints

The defecation frequency, stool volume, stool consistency and the number of Rome III positive criteria between the two groups did not differ at baseline. After the intervention period, there were significant increases in defecation frequency in both groups compared to baseline (RMD 65.8 %, placebo 56.1 %; *p* < 0.01 each). By contrast, the stool volume evolved differently between two groups. There were significant increases found in the RMD group at Week 1, 2, and 3 (31, 32, 56 %; *p* < 0.006, *p* < 0.006, *p* < 0.0001, respectively) compared to baseline, while in the placebo group there were no changes found during intervention (Table 3). Furthermore, there was a significant time-by-treatment interaction observed in stool volume (*p* = 0.014). Based on the Bristol Scale Score self-recorded, a significant increase (softer and better stool consistency) was observed in the RMD group after 3 weeks (21.4 %, *p* < 0.01) compared to baseline. No change was observed in the placebo group for the stool consistency. Regarding the total number of Rome III positive criteria, there were significant reductions in both RMD and placebo groups (*p* < 0.01 each).

**Table 3:** Change in stool volume and stool consistency for each group during 3-week intervention

	Baseline	Week 1	Week 2	Week 3	*p* value^†^ time × treatment
Stool volume (cm^3^/day)					
RMD	53.4 ± 30.1	69.9 ± 27.4*	70.2 ± 27.3*	83.4 ± 26.6*	0.014
(*Δ*)		(16.6 ± 32.9)	(16.8 ± 31.2)	(30.0 ± 27.4)^##^	
Placebo	56.4 ± 20.6	59.4 ± 25.0	64.3 ± 25.1	65.6 ± 23.8	
(*Δ*)		(3.0 ± 21.4)	(7.9 ± 24.8)	(9.2 ± 20.0)	
Stool consistency (score)					
RMD	2.8 ± 1.2	3.0 ± 1.0	3.0 ± 1.2	3.4 ± 1.0*	n.s.
(*Δ*)		(0.3 ± 1.0)	(0.3 ± 1.2)	(0.6 ± 0.8)	
Placebo	2.8 ± 0.8	3.2 ± 0.8	3.2 ± 0.7	3.2 ± 0.5	
(*Δ*)		(0.4 ± 1.0)	(0.4 ± 0.9)	(0.4 ± 0.8)	

Values expressed as mean ± SD (standard deviation

*Δ*: change from baseline to each time point; n.s.: no significant

* *p* < 0.05, intragroup significant difference (Bonferroni)

^†^ *p* for time-by-treatment interaction was assessed by a repeated-measures ANOVA

^##^ *p* < 0.01, intergroup significant differences (ANOVA)

Interestingly, analysis of the individual Rome III criteria showed that only after intervention with RMD a significant lower number of subjects (compared to baseline) answered positively to 3 out of 6 criteria: “straining” (from 63.6 to 33.3 %; *p* < 0.025), “sensation of incomplete evacuation” (from 51.5 to 27.3 %; *p* < 0.003) in at least 25 % of defecations, as well as “fewer than three defecations per week” (from 24.2 to 0.0 %; *p* < 0.013).

### Safety

During the study, six AEs were recorded (2 in placebo and 4 in RMD groups). None of the AEs were related to the study products nor classified as serious. These events were: ankle sprain, cervical muscle spasm, otitis, fever and diarrhoea. There were no clinically relevant changes observed in the biochemical parameters by haematological examination.

## Discussion

This randomized, placebo-controlled, double-blind study demonstrated that daily consumption of 15 g of a RMD supplementation for 21 days significantly reduces the total CTT in healthy subjects. The 25.1 % reduction in the total CTT was primarily due to the 31.5 % reduction in the left CTT. This reduction is physiologically relevant since the total and segmented CTT at baseline was within the values previously reported for healthy individuals in other studies [33, 34]. Some reports have been shown the gender differences [35], and others not [36]. There was no gender difference in this study. The lack of gender-related CTT differences can be accounted for by the young age range of the healthy participants [34]. Subject’s loss in measurement of CTT variable for failure to measurement requirements did not affect the outcome since the loss was homogeneous in both groups. Additionally, the results presented herein are consistent to demonstrate that the intake of RMD provokes increased stool volume, better stool consistency and improvement in the intestinal functions. The consumption of daily RMD also demonstrated some more benefits to reduce the frequency of straining and sensation of incomplete evacuation. These results are consistent with the widely reported beneficial effects of DF intake [37, 38].

To our knowledge, there were no reports that confirmed a significant reducing effect on CTT in humans with other soluble DFs (SDFs) before the current study with RMD. For example, using either 15 or 20 g/day of inulin for 21 days did not influence CTT [39, 40]. Another study reported that 8 g/day of fructo-oligosaccharides (FOS) for 28 day failed in modifying CTT, and a meta-analysis of FOS concluded that there is no reducing effect on CTT [41, 42]. Among other studies of SDF, using 3.4 g/day of pectin for 28 days, 6 g/day of pectin for 21 days [43, 44], 15 g/day of guar gum for 18 days [45], 20 g/day of soluble corn fibre for 10 days [46], either 20 or 30 g/day of polydextrose for 10 days [46, 47] and 30 g/day of arabinogalactan for 21 days [48], no changes on CTT have been observed. One study with 8 g/day polydextrose in 100 g yogurt product reported to show a significant change to shorten CTT after 21-day administration; however, a significant change was also found in the placebo group, indicating that it was an effect due to the yoghurt product [49]. The current study suggests that even in the same category of SDF, the effect on the CTT is different. One of the reasons could be the characteristics that each SDF possesses such as fermentability by the intestinal bacteria and/or the presence or absence of increasing stool weight.

Faecal matter is transported from the ascending colon (right colon) to the descending colon (left colon) by intestinal peristalsis. The peristaltic motion is caused by gastrocolic reflex that occurs when food enters the stomach and in response to physical stimuli by the stool bulk. In addition, it has been reported that short-chain fatty acids (SCFAs) produced by the intestinal bacteria could also stimulate the peristalsis [50]. When ingested orally, about 10 % of RMD is digested and absorbed as glucose in the small intestine, while remaining 90 % reaches the large intestine. Some portion of RMD that reaches the colon is fermented slowly by the intestinal bacteria such as bifidobacteria, and the portion that escapes from bacterial fermentation is excreted with faeces. It has been confirmed that the intake of RMD leads fermentation by intestinal bacteria and production of SCFA in humans [23, 25]. The ratio between the amount fermented and excreted has been reported as approximately 50–50 although it could vary between individuals [24, 30]. The fact that a part of RMD is excreted with the faeces leads to an increase in stool volume [51, 52]. Consequently, it is considered that the increased amount of stool bulk and the production of SCFA stimulate the intestinal peristalsis, contributing to the reduction in CTT.

The effect of RMD to reduce the CTT in humans was confirmed in the present study, and this effect could deeply associate with the characteristics of RMD. A distinctive characteristic of RMD is a slow speed of fermentation in the large intestine compared to other SDF. According to the previous report, the estimated speed of fermentation is FOS > guar gum > RMD [53]. Another report showed that inulin is faster than guar gum [54]. Therefore, FOS, inulin and guar gum are rapidly fermented and consumed in the first half of the colon, i.e. ascending colon on the right side, before reaching the second half of the colon. On the other hand, the slow and stable fermentation of RMD enables it to reach the second half of the colon, i.e. descending colon on the left side, meaning that it provides a source of fermentable carbohydrate to the more distal part of the large intestine. The SCFA produced by fermentation reportedly cause spontaneous contraction of colon [50]. Thus, it is considered that SCFA produced by RMD in the second half of the large intestine would induce the intestinal peristalsis, resulting in significant reductions in the left CTT and rectosigmoidal CTT. Consequently, the total CTT is shortened significantly. Likewise, there is also the possibility that the effect of RMD to increase stool bulk generates a synergistic effect with the effect of producing SCFA, which brought out the remarkable result observed after the RMD intake.

One limitation in this study could be self-reporting nature for defecation variables. However, it is important to note that this limitation could be more relevant in some variables than in others. For instance, the effect to increase stool weight and volume by the intake of RMD has been reported in the multiple human studies that evaluated this variable using either the measurement of stool weight excreted [55, 56] or the visual scoring method [51, 52]. The result by visual evaluation has been reported to have a high correlation with the result by weight measurement, suggesting that besides of being a simple method, the visual method is reliable [57]. By contrast, the defecation frequency could have unintentionally been increased by the subject in the placebo group, since they knew this study assessed intestinal functions. Thus, defecation frequency is easily the most evident effect that subjects could expect from the intervention and so feeling subconsciously the need to have more bowel movements. The aforementioned observation explains why defecation frequency in the placebo group was the only variable that displayed a significant change. The strengths of the present study included the reliable technique used to assess the primary endpoint. In order to obtain accurate CTT in humans, the study method is extremely important. Since Hinton et al. [58] introduced a method using radiopaque marker, it has become the standard to ingest radiopaque markers for CTT evaluation. The method, however, requires collection of faecal samples for several days to determine by X-ray, and its limitation has been pointed out that the determination of CTT based on the 80 % recovery in the faeces could cause a margin of error. A new method was proposed to perform a single abdominal X-ray of subjects after every 24-h consecutive ingestion of radiopaque markers, providing a less demanding procedure for subjects to finish by only one visit for X-ray test without faecal collection. Additionally, the method provides the data of not only CTT but also SCTT in the large intestine. In this study, a single X-ray method was used based on the method by Bouchoucha et al. to minimize radiation exposure and to obtain reliable data of CTT and SCTT [19]. As a result, CTT before and after the ingestion of placebo was almost the same, suggesting that the study to determine CTT was conducted accurately with good management of subjects and that the results have high reliability.

Although this is the first study for RMD to evaluate the efficacy on CTT in Western population, the supplementation of 15 g/day made up for a gap to fulfil 25 g of DF as recommended daily intake for adult in EU and other countries worldwide. Soluble RMD has physiological benefits and advantage as easy-to-use in food applications or as a supplement. Dietary supplementation of RMD has the possibility to help improving DF deficit in the countries, where people tend to consume more processed foods.

## Conclusions

The results demonstrate that the supplementation of RMD has a beneficial effect in improving colonic transit time, stool volume, stool consistency and some clinical intestinal functions (for instance, the straining and sensation of incomplete evacuation), in a healthy young Western population. Furthermore, RMD supplementation has shown to have an adequate safety profile. The results open the possibility for the further research of RMD in patients suffering from gastrointestinal disorders in Western populations.
